# From Bench to Bedside: Immunotherapy for Prostate Cancer

**DOI:** 10.1155/2014/981434

**Published:** 2014-09-04

**Authors:** Brian Wan-Chi Tse, Lidija Jovanovic, Colleen Coyne Nelson, Paul de Souza, Carl Andrew Power, Pamela Joan Russell

**Affiliations:** ^1^Australian Prostate Cancer Research Centre-Queensland, Institute of Health and Biomedical Innovation, Queensland University of Technology, Translational Research Institute, 37 Kent Street, Brisbane, QLD 4102, Australia; ^2^Sydney School of Medicine, University of Western Sydney, Ingham Institute for Applied Medical Research, Campbell Street, Sydney, NSW 2170, Australia; ^3^Biological Resources Imaging Laboratory, Lowy Cancer Research Centre, University of New South Wales, High Street, Sydney, NSW 2052, Australia

## Abstract

The mainstay therapeutic strategy for metastatic castrate-resistant prostate cancer (CRPC) continues to be androgen deprivation therapy usually in combination with chemotherapy or androgen receptor targeting therapy in either sequence, or recently approved novel agents such as Radium 223. However, immunotherapy has also emerged as an option for the treatment of this disease following the approval of sipuleucel-T by the FDA in 2010. Immunotherapy is a rational approach for prostate cancer based on a body of evidence suggesting these cancers are inherently immunogenic and, most importantly, that immunological interventions can induce protective antitumour responses. Various forms of immunotherapy are currently being explored clinically, with the most common being cancer vaccines (dendritic-cell, viral, and whole tumour cell-based) and immune checkpoint inhibition. This review will discuss recent clinical developments of immune-based therapies for prostate cancer that have reached the phase III clinical trial stage. A perspective of how immunotherapy could be best employed within current treatment regimes to achieve most clinical benefits is also provided.

## 1. Introduction

Prostate cancer (PCa) is the most frequently diagnosed cancer in men and the sixth leading cause of death from cancer in men worldwide [1]. An estimated 903,000 men were newly diagnosed, and 258,000 died from PCa in 2008 [1]. When diagnosed at its early stages, PCa can be effectively treated by surgery or radiation. However, up to one-third of patients with organ-confined PCa eventually fail local therapy and ultimately progress to advanced-staged or metastatic disease within 10 years [2]. Approximately 4% of all newly diagnosed patients present with metastatic disease, and up to 85% of patients diagnosed with CRPC have metastases [3].

## 2. Current Therapeutic Options for PCa

The normal prostate and PCa require androgens for growth and optimal function of cell survival pathways [4]. After local surgery or radiation, cancer control is monitored by Prostate Specific Antigen (PSA), a prostate epithelial-specific protein detected from secretions into the blood stream. The suppression of androgens by androgen deprivation therapy (ADT) alone or together with androgen receptor (AR) antagonists initially induces tumour regression and a period of cancer control, accompanied by nondetectable or exceedingly low PSA levels [5]. Ultimately, patients relapse, signalled by a rise in PSA, and develop CRPC. Docetaxel (Taxotere) was the first chemotherapy drug to show improved survival for patients with CRPC, compared to the then standard of care, mitoxantrone [6, 7]. More recently, cabazitaxel was FDA-approved for patients who fail docetaxel therapy on the basis of prolonged survival [8]. The seminal finding that PCas can undertake* de novo* steroidogenesis and synthesis of androgens and other steroids that reactivate the AR [9] has underpinned the rationale for developing the steroid synthesis CYP17A1 inhibitor, abiraterone; this was approved for clinical use after landmark trials showing extended survival after docetaxel failure [10, 11]. The new AR antagonist, enzalutamide, is the latest drug to be FDA-approved in CRPC, again on the basis of prolongation of survival compared to placebo [12]. Cancer immunotherapy has recently been introduced into the therapeutic landscape for CPRC following the approval of the dendritic-based cancer vaccine sipuleucel-T (Provenge) by the FDA in 2010 [13]. The goal of immunotherapy is to harness the powerful capabilities of the immune system, comprising both the adaptive and innate arms, to effectively recognise and kill transformed cells whilst sparing healthy tissues. For excellent reviews on the mechanisms of antitumour immunity induction, see [14–16]. Currently, various forms of immunotherapy are being investigated in clinical trials for PCa including dendritic cell-based vaccines, immune checkpoint inhibition, viral-based vector, and whole cell-based vaccines.

## 3. Immunotherapy Is Rational for PCa Treatment

### 3.1. Androgen Deprivation and Immune Changes

Over the past decade, strong evidence that PCa is inherently immunogenic has emerged, which underpins the rationale for using immune-based therapies for this disease. PCa can stimulate immune responses, as evidenced by induction of T cell responses to cancers by various immunotherapies (see later sections), and by histological data revealing the presence of CD4+ T cells, CD8+ T cells, natural killer (NK) cells, dendritic cells, and macrophages within tumours. Early studies reported that prostate tumours with a dense infiltration of lymphocytes correlated with longer patient survival and that high grade prostatic adenocarcinomas have significantly less infiltration of T cells and macrophages as compared to benign nodular prostatic hyperplasia [25, 26], suggesting that tumour progression could be associated with defects in cell-mediated immune responses. Although subsequent studies found that greater tumour infiltration of CD4+ T cells can predict poorer prognosis [27], we now appreciate that a proportion of these were likely regulatory T cells (Tregs), which suppress immune responses through their inhibitory actions at both the induction and effector phases. High tumour infiltration of forkhead box P3- (foxp3-) expressing cells (Tregs) was also found to correlate with higher baseline PSA levels [28]. A high prevalence of regulatory T cells within tumours is associated with more lethal PCa [29], suggesting that therapeutic blockade of these cells may induce beneficial clinical responses, by facilitating the generation of effective cytotoxic immune responses. Increased NK cell infiltrate within tumours was also found to be associated with a lower risk of progression, providing evidence that these innate immune cells may have a protective role against PCa in humans [28]. A recent study reported that in organ-confined PCa, the prevalent macrophage phenotype was M1 (tumour-inhibitory, promotes Th1 responses, proinflammatory; defined as CD68+ by those authors) whereas in PCas with extracapsular extension, M2 macrophages (tumour-promoting, proangiogenic, promotes Th2 responses; defined as CD163+) were more prevalent [30]. These findings, together with another observation of reduced infiltration of CD68+ macrophages associated with lymph node positivity and higher clinical stage [31], indicate that reduced numbers of macrophages with cytotoxic capabilities parallel more aggressive disease; hence immune interventions that shift the balance of macrophages to the M1 phenotype may have antitumour effects. Despite this, it is important to note that other studies have reported infiltration of CD68+ macrophages to correlate with poorer patient outcomes [28, 32]. While there is strong evidence that PCa is immunogenic, it is also known that these cancers employ numerous immune escape strategies that contribute to their growth and progression. Such escape mechanisms include defects in antigen presentation (e.g., downregulation of HLA class I antigens and beta-2 microglobulin to escape killing by cytotoxic T cells), induction of T cell death (e.g., expression of Fas ligand to trigger apoptosis of Fas-expressing immune cells), production of immunosuppressive cytokines (e.g., transforming growth factor-*β*), and recruitment of regulatory T cells, which limit immune responses [33]. These are challenges that immunotherapies need to overcome, in order to deliver clinical benefits to PCa patients.

Realistically, if immunotherapies are to be incorporated into treatment regimens for PCa, they would most likely be employed as adjunct therapies to ADT, the mainstay approach for both high-risk early PCa and recurrent or metastatic disease. Further evidence in support of the rational use of immune-based therapies for PCa is that ADT seems to enhance the influx of immune cells into tumours. Androgen-depleted patients were reported to have a 5-fold increase in CD4+ T cell infiltration, 2-fold increase in CD8+ T cell infiltration, and 3-fold increase in macrophages, as compared to untreated tumours [34]. Moreover, hormone ablation resulted in an increase in the number of tumour-resident cells expressing the costimulatory molecules B7.1 and B7.2, which are necessary for effective T cell activation [34]. A significant proportion of T cells from patient tumours expressed IFN*γ*, the proliferation marker Ki67, and exhibited a restricted pattern of T cell receptor (TCR) beta-chain variable region (V*β*) gene usage, an oligoclonal response consistent with an antigen-specific response [34]. Similar findings were demonstrated in another report whereby androgen-depleted tumours had a greater density of total T cells, as well as the CD8+ T cell subset [28]. These results suggest that ADT may augment antitumour immunity by enhancing T cell activation as well as their trafficking to tumours. The relative timing of androgen ablation with immunotherapy would be crucial and warrants investigation.

### 3.2. Tumour Associated Antigens and Their Role in the Immune Response

The existence of several tumour-associated antigens (TAAs) provides further evidence in support of the view that PCa would be amenable to immunotherapy; these include the serine protease prostate specific antigen (PSA; kallikrein 3), prostatic acid phosphatase (PAP), prostate specific membrane antigen (PSMA), prostate stem cell antigen (PSCA), mucin-1 (MUC-1), and NY-ESO-1 [2]. Although in a steady state these self antigens do not provoke strong immune responses, due to multiple layers of tolerance mechanisms, nevertheless, several immunotherapeutic strategies have been shown to induce or enhance responses to these antigens [2]. PSA, in addition to being selectively expressed by prostate epithelial cells, is also a well-characterised serum biomarker for PCa progression; therefore it also has utility in the assessment of patient treatment response. Another characteristic of PCa which renders it attractive for immunotherapy is that it is a relatively slowly progressing disease, allowing sufficient time for the immunologic intervention to take effect. In addition, the prostate gland is nonessential for life; therefore even if the immune response destroys normal prostatic tissue, it would not be life-threatening or likely to cause significant morbidity. Thus, immunotherapy is a rational approach for PCa treatment. This review will discuss recent clinical development of immune-based therapies for PCa that have reached the phase III clinical trial stage, to highlight the range of strategies currently being explored and that show promise. These are sipuleucel-T (dendritic cell-based vaccine), ipilimumab (immune checkpoint inhibitor), Prostvac-VF (viral-based vaccine), and GVAX (whole cell-based vaccine) (Table 1).

## 4. Sipuleucel-T (Dendritic Cell-Based Vaccine)

The rationale for using autologous dendritic cells in cancer immunotherapies is based on their efficient activation of antigen-specific cytotoxic T cells to kill cancer cells. Sipuleucel-T is a dendritic-based immunotherapy in which autologous peripheral blood mononuclear cells (PBMCs) are incubated* ex vivo* for 36–48 hours with a fusion protein (PA2024) of PAP and granulocyte-macrophage colony-stimulating factor (GM-CSF) [35]. An analysis of culture supernatant during the manufacture process showed an increase in antigen presenting cell (APC) activation cytokines (interleukin (IL)-1a, IL-23, macrophage inflammatory protein (MIP)-1a and -1b), T cell activation markers (IL-2, -3, -4, -5, -10, and -17) as well as APC/T cell activation-associated cytokines (IL-12, IFN*γ*, tumour necrosis factor (TNF)) [36]. After sipuleucel-T treatment of autologous cells, the product containing increased activated APCs and T cells is reinfused into the patient and contains at least 50 million autologous activated CD54+ dendritic cells, and a variable number of T cells, B cells, natural killer cells, and others [37]. Sipuleucel-T immunotherapy targets cells which express PAP, a secreted glycoprotein enzyme that is expressed in 95% of prostate tissue and PCa; expression levels correlate with tumour staging [38, 39]. Moreover, high serum PAP levels are associated with significantly shorter survival and lower responsiveness to radiation therapy [40]. The GM-CSF component of the fusion protein is an immune modulatory cytokine that stimulates the development and maturation of APCs, including type 1 dendritic cells (DC1), the subset responsible for initiation of cytotoxic immune responses [41]. A key preclinical study which laid the groundwork for the clinical development of sipuleucel-T demonstrated that mice immunised with APCs incubated with a fusion protein of rat PAP and rat GM-CSF developed profound prostatitis (marked lymphocyte infiltrate), indicating that this approach could break tolerance to the self-antigen [42, 43]. In a follow-up experiment, coculture of PAP-expressing tumour cells with splenocytes from animals immunised with APCs pulsed with PAP and GM-CSF inhibited tumour cell proliferation [42], showing the potential antitumour effect of the cancer vaccine. These preclinical studies formed the basis for clinical trials of sipuleucel-T.

In a sequential phase I/II clinical trial involving 31 men with progressive disease despite ADT (12 with metastatic disease in the phase I cohort and 19 with nonmetastatic disease in phase II), sipuleucel-T was found to be well-tolerated, with preliminary evidence of clinical efficacy [44]. 100% patients developed T cell proliferative responses to the fusion protein, PA2024, and 38% against native PAP, following sipuleucel-T administration (none had preexisting responses to either antigen), suggesting that immunological tolerance against PA2024 and PAP could be broken by this therapy [44]. Importantly, patients that developed an immune response against PAP, whether in T cell proliferation or antibody development, had significantly longer median time to disease progression as compared to those who did not (34 versus 13 weeks, resp.; *P* < 0.027) [44]. In total, 6 patients had PSA value declines of greater than 25% from baseline. In another phase I clinical trial of 13 patients with similar clinicopathological characteristics, sipuleucel-T was again well-tolerated, and all patients developed measurable antigen-specific T cell responses and antibodies against PA2024 after immunisation [45].

These promising findings led to the commencement of three phase III clinical trials, all of which have now been completed. In the first study (D9901), 127 patients with asymptomatic metastatic CRPC were assigned in a 2 : 1 ratio of sipuleucel-T (*n* = 82) or placebo (infusion of APCs not pulsed with PA2024; *n* = 45) [17]. While there was no difference in the primary endpoint of time to progression (TTP) between the 2 treatment groups (11.7 weeks for sipuleucel-T and 10.0 weeks for placebo), there was a significant increase in the secondary endpoint of overall median survival for those given sipuleucel-T (25.9 months versus 21.4 months) [17]. Moreover, the survival rate at 36 months was significantly higher (*P* = 0.005) for sipuleucel-T treated patients (34%) when compared to placebo (11%). The clinical efficacy of this dendritic-based immunotherapy was confirmed in another similarly designed clinical trial of 98 patients (65 for sipuleucel-T and 33 for placebo) (D9902A) [18]. No difference in TTP was observed between the two groups, although the median survival time was greater for sipuleucel-T-treated patients over placebo (19.0 months versus 15.7 months, resp.), consistent with the prior clinical trial [18]. The 36-month survival rates were 33% (sipuleucel-T) and 15%, (controls). A third phase III clinical trial known as the Immunotherapy for Prostate Adenocarcinoma Treatment (IMPACT) trial was undertaken; 512 patients were involved (341 on sipuleucel-T and 171 on placebo) [19]. Consistent with previous trials, sipuleucel-T treatment provided a 4.1 month improvement in median survival (25.8 months versus 21.7 months), and the 36-month survival rate was 31.7% versus 23.0% [19]. The time to disease progression was similar between the treatment groups. Interestingly, multivariate analysis showed that baseline PSA levels provided a strong prognostic factor, whereby the overall survival hazard ratio in the lowest baseline PSA quartile (<22.1 ng/mL) was 0.51 (0.31–0.85) compared with the highest PSA quartile (<134 ng/mL) of 0.84 (0.55–1.29) [46]. This finding suggests that patients with lower tumour burden are more likely to benefit most from sipuleucel-T and provides a rationale to employ this immunotherapy as an early treatment strategy. Since the 3 aforementioned phase III clinical trials were similarly designed, and recruited patients with similar characteristics, an analysis for immune parameters with overall survival was performed on pooled data (*n* = 737) [36]. Overall survival significantly correlated with development of at least one postbaseline peripheral immune response (T cell proliferation, IFN*γ* ELISPOT, or antibody production) to PA2024 or PAP (HR = 0.47) (95% CI: 0.29–0.78) [36]. In sipuleucel-T treated patients, a positive correlation between overall survival and cumulative APC activation (increase in CD54 expression on APCs; from pre- to postculture with the fusion protein), APC count and total nuclear cell (TNC) count were observed [36]. Whilst these studies have collectively confirmed the clinical utility of sipuleucel-T for the treatment of advanced PCa, some questions remain as to why progression-free survival is unaffected. One possible explanation relates to how progression is defined, which in these studies is an increase in measurable disease based on radiographic imaging, new cancer-related pain associated with radiographic correlation, or other clinical events consistent with progression such as spinal cord compression or pathological fracture. The development of new lesions could have been interpreted as progression yet the disease was responding to treatment. Another speculation is that the treatment gradually slows down progression, which is reflected in prolongation of overall survival, but short-term improvements are not apparent. In addition, since the mode of action of Sipuleucel-T is to activate the immune system, a delay in treatment effect is expected. It is also important to note that PSA levels generally do not alter in response to Sipuleucel-T, further highlighting the difficulty in assessing the therapeutic response.

## 5. Ipilimumab (Immune Checkpoint Inhibitor)

T cell responses are initiated through the interaction of the T cell receptor (TCR) with antigen presented in major histocompatibility complex (MHC) molecules on antigen presenting cells (APCs). However, the quality and magnitude of the response are dependent on a fine balance of costimulatory and coinhibitory signals delivered to the T cell; for an excellent review on T cell costimulation see [47]. CD28 (a costimulatory receptor) and cytotoxic T-lymphocyte-associated antigen 4 (CTLA-4) (a coinhibitory receptor) are homologues that compete for their ligands, CD80 (B7-1) and CD86 (B7-2) expressed on APCs [47]. CTLA-4 is a key negative regulator of T cell responses and is upregulated following T cell stimulation to attenuate the response. It therefore functions as an immune checkpoint molecule. CTLA-4 is also constitutively expressed on regulatory T cells and mediates their immune suppressive effects [48]. From a therapeutic point of view, CTLA-4 blockade could prevent immune inactivation, leading to robust antitumour immune responses. In a preclinical model of PCa,* in vivo* blockade of murine CTLA-4 with an antibody significantly delayed the growth of subcutaneous TRAMP-C1 tumours [49], derived from Transgenic Adenocarcinoma of the Mouse Prostate (TRAMP) mice [50, 51] which spontaneously develop PCas due to prostate-specific expression of Simian virus 40 (SV40) large T antigen (Tag). In a follow-up study, CTLA-4 blockade prevented and/or slowed the outgrowth of subcutaneous TRAMP-C2 tumours, which spontaneously metastasise to the regional lymph nodes [52]. Moreover, anti-CTLA-4 antibody treatment after surgical resection of the primary tumour facilitated the elimination of lymph node metastases, suggesting that this immunotherapy has potential to be used as an adjunct therapy to eliminate residual metastatic PCa after surgery [52]. Based on these preclinical studies, clinical trials of CTLA-4 blockade for PCa treatment have been initiated.

Ipilimumab is a fully human IgG1 monoclonal antibody that binds to and blocks the activity of CTLA-4. It was approved by the FDA in 2011 for the treatment of advanced melanoma on the basis of prolonged survival [53, 54]. Ipilimumab is currently being trialled for the treatment of non-small cell lung cancer (NSCLC) [55], metastatic renal cell cancer [56], ovarian cancer [57, 58], and PCa. In a phase I clinical trial comprising 14 patients with progressive metastatic CRPC (some of whom previously also received chemotherapy or other investigational drugs), ipilimumab treatment at 3 mg/kg was well tolerated without the development of significant pathological autoimmunity [59]. A trend observed across the cohort was a gradual increase in the percentage of CD4+ HLA DR+ T cells in peripheral blood following ipilimumab treatment [59], consistent with its mode of action in modulating immune activation. Two of 14 patients also had a >50% decline in PSA, which lasted for 135 and 60 days, respectively. In another phase I/II clinical trial involving a similar cohort of PCa patients, ipilimumab was administered alone or in combination with radiotherapy (8 Gy per bone target, up to 3 lesions per patient) [60]. The maximal tolerable dose was determined as 10 mg/kg, with common immune-related adverse events such as diarrhoea, colitis, and rash usually of grade 1/2 [60]. For patients that received this dosage (with or without radiotherapy) (*n* = 50), eight had PSA declines of >50%, one had complete response, and six had stable disease. These observations indicated potential clinical antitumour activity of ipilimumab and led to a phase III clinical trial that compared ipilimumab against placebo after radiotherapy of mCRPC patients who have failed docetaxel (*n* = 799) [20]. While there was no significant difference in terms of overall survival (primary endpoint) between the ipilimumab (11.2 months; 95% CI 5–12.7) and placebo (10 months; CI 8.3–11) groups, there were signs of activity with the drug [20]. Progression-free survival at 6 months was 30.7% (95% CI 26–35.3) for ipilimumab, and 18.1% (14.3–22) for placebo. A decline in PSA levels (>50% at any time) was also more frequent with the ipilimumab group (13.1%; 9.5–17.5) than placebo group (5.2%; 3–8.4). However, an exploratory and post-hoc subgroup analysis showed overall survival benefit for ipilimumab after radiotherapy in a subset of patients with favourable prognostic features (no visceral metastases, nonraised or mildly raised alkaline phosphatase, and without anaemia). The median overall survival was 22.7 months for ipilimumab (*n* = 146), as compared to 15.8 months for placebo (*n* = 142). This apparent benefit from ipilimumab in patients with lower tumour burden warrants further investigations, as it has significant implications for further treatment studies using ipilimumab for this disease.

## 6. Prostvac-VF (Viral-Based Vaccine)

Viral vectors are attractive for use in cancer immunotherapies as they can mimic natural infection and lead to the induction of robust immune responses against the tumour antigen(s) that they encode [61]. Prostvac-VF (or PSA-TRICOM) is a recombinant viral vaccine currently being trialled as an immunotherapy for PCa that involves a prime and multiple booster injections with attenuated strains of* Vaccinia* and* Fowlpox *viruses, respectively [62]. Both recombinant viruses are engineered to encode the entire PSA gene with a modified agonist epitope (increases immunogenicity) and 3 costimulatory proteins: B7-1 (facilitates T cell activation), lymphocyte function-associated antigen 3 (LFA-3; CD58), and intercellular adhesion molecule-1 (ICAM-1; CD54) (both are cell adhesion molecules that strengthen interactions between APCs and T cells). The rationale behind this approach is that the virus will directly infect the APCs (resulting in expression of the costimulatory molecules), or somatic cells (epithelial and/or fibroblasts) at the site of injection, leading to cell death and subsequent uptake of cellular debris containing PSA by the APCs [63]. The transduced APCs, or antigen-loaded APCs, upon interaction with CD4+ and CD8+ T cells, will effectively promote the development of T cell-mediated immune responses that destroy PSA-expressing cancer cells. The viral vectors are nonreplicative and do not integrate into the genome of host cells, thus allowing for controlled administration of the transgenes [62]. Sequential injection of the* Fowlpox*-based booster vaccine is designed to overcome the reduced immunisation response following the development of neutralising antibodies against the* Vaccinia*-based priming vaccine, so that a level of immunity is maintained. Several preclinical studies have shown that the 3 costimulatory molecules, commonly referred to as TRI-COM, act synergistically to enhance antitumour immune responses to tumour self-antigens [64]. For example, vaccination of transgenic mice expressing human carcinoembryonic antigen (CEA-Tg) with recombinant* Vaccinia* and* Fowlpox* vectors encoding for CEA and TRI-COM can facilitate rejection of CEA-expressing murine colon carcinomas, an effect dependent on CD4+ and CD8+ T cells but not NK cells [64]. These mice had a longer survival time and did not develop pathologic autoimmunity based on biochemical, immunological, and histological criteria, demonstrating the therapeutic potential of this vaccine approach with minimal toxicity. Such studies have formed the basis for the development of Prostvac-VF.

A number of phase I clinical trials have established the safety and tolerability of Prostvac-VF [65, 66], and a phase II study was used to optimise the sequence of vector administration (one* Vaccinia *prime followed by three* Fowlpox *boosts) [67]. These findings led to the commencement of a phase II randomised controlled trial of 125 patients with minimally symptomatic CRPC (82 given Prostvac-VF and 40 control empty viral vectors) [21]. Prostvac-VF treatment was associated with longer overall survival time (25.1 months) as compared with control (16.6 months)—an improvement in median survival of 8.5 months [21]. The 3-year survival rate was almost double (30.5% versus 17.5%). However, no difference in progression-free survival was observed between the 2 arms (primary endpoint). Consistent with these findings, another phase II trial (nonrandomised) reported that Prostvac-VF treatment correlated with improved overall survival as compared with historical controls (the Halabi nomogram) (26.6 versus 17.4 months); the Halabi nomogram is a prognostic model developed on historical data that is designed to predict survival of mCRPC patients given docetaxel [22]. Importantly, patients with a Halabi who predicted survival of <18 months had an actual overall survival median of 14.6 months following Prostvac-VF treatment, whereas for those with a Halabi prediction of ≥18 months, it was ≥37.3 months (not yet reached) [22]. This suggests that patients with less aggressive or earlier-stage disease are more likely to benefit most from this vaccine; this could influence how Prostvac-VF may be employed clinically. Moreover, this study showed that 12 of 32 evaluable patients (37.5%) showed a decline in serum PSA after vaccination, and 2 of 12 patients with soft tissue metastases exhibited a measurable decrease in index lesions by CT [22]. Recently, an analysis of immunological impact by Prostvac-VF on pooled data from several clinical trials conducted similarly was published [68]. Collectively, 59 of 104 patients (57%) had an increase in the number of PSA-specific T cells following vaccination (median of 5-fold), and interestingly, 19 of 28 patients (68%) exhibited evidence of immune responses mounted against other tumour-associated antigens not found in the vaccine, for example, MUC1; a phenomenon known as antigen spreading [68]. This could be another mechanism that mediates the antitumour effect of Prostvac-VF. Moreover, the number and function of regulatory T cells were deceased following vaccination, although no effect on NK cell numbers was seen [68]. Due to these promising results, Prostvac-VF is currently being investigated in phase III clinical trials.

## 7. GVAX (Whole-Cell Based Vaccine)

GVAX is a whole-cell vaccine comprised of a mixture of 2 human PCa cell lines, LNCaP (androgen-sensitive; derived from a lymph node metastasis) and PC3 (androgen-insensitive; derived from a bone metastasis), which are modified to constitutively express GM-CSF and irradiated to prevent cell replication [69]. The rationale behind this vaccine approach is to mount immune responses against shared tumour antigens between the two allogeneic cell lines and the host cancer, with GM-CSF enhancing this process by functioning as a chemoattractant for dendritic cells as well as facilitating their maturation and antigen-presentation to T cells to elicit robust antitumour effects [69]. The use of allogeneic tumour cells as the core component also has advantages in being faster and less expensive to manufacture as compared to autologous tumour cells, which are technically more difficult to harvest. Several preclinical studies have demonstrated that this whole-cell vaccine approach can induce potent antitumour effects in a number of cancer types. For example, subcutaneous injection of irradiated murine B16 melanoma cells retrovirally modified to express GM-CSF was associated with lower tumour take rate and longer survival time as compared to nontransduced cells, an antitumour effect mediated by both CD4+ and CD8+ T cells [70]. In addition, mice injected with irradiated GM-CSF-expressing tumour cells rejected a subsequent challenge with nontransduced B16 cells, but not Lewis lung carcinoma cells, indicating the vaccination induced strong protective tumour-specific immune responses [70]. Similar results were found in a preclinical model of PCa, whereby rats implanted with irradiated MatLyLu prostate carcinoma cells engineered to express GM-CSF exhibited longer disease-free survival compared to those challenged with nontransduced cells but also received soluble GM-CSF [71], indicating that the continual tumour secretion of this cytokine facilitates the development of antitumour immunity. These early studies collectively formed the foundation for clinical trials of GVAX for PCa.

A number of phase I/II clinical trials have demonstrated the safety and tolerability of GVAX [72]. Early evidence for clinical responses was observed in a phase II trial involving 55 men with chemotherapy-naive metastatic CRPC; 34 with radiologic metastases (24 with high dose GVAX and 10 with low dose); and 21 with rising PSA (all low dose) [73]. In the radiologic group, the median time to PSA progression was 2.3 months for those given low dose, 3.7 months for high dose, and for the PSA-rising group, it was 3.9 months [73]. The overall median survival time after treatment initiation was 24 months (low dose) and 34.9 months (high dose) in the radiologic group, whereas the estimated media survival of these patients based on the Halabi nomogram was 19.5 months, showing a trend of increased survival time by GVAX in a dose-dependent fashion. Moreover, there was evidence for induction of antibodies to PC3 cell lysate (18/28; 64.3%) and LNCaP (12/28; 64.3%) by patients in the radiologic group, as shown by immunoblots with patient sera. In another phase II clinical trial comprised of 80 men with the same clinicopathological characteristics, treatment with high dose was associated with longer median survival time (35 months) as compared with those given medium dose (20 months) and low dose therapy (23.1 months) [74]. The proportion of patients that generated an antibody response to either cell line was 89%, 72%, and 43%, respectively [74]. Collectively, those who exhibited antibody induction had a median survival of 34 months (*n* = 30), compared to 16 months for those who did not (*n* = 6), suggesting that immune reaction is associated with better clinical outcomes. These promising results led to the two phase III clinical trials which recruited men with metastatic CRPC comparing GVAX with docetaxel-prednisone with (trial named VITAL-1) or without (VITAL-2) cancer-related pain [75]. Unfortunately, VITAL-2 (*n* = 408) was terminated early as preliminary analysis demonstrated an excessive number of deaths (67 versus 47) and shorter median survival (12.2 months versus 14.1 months) for GVAX and docetaxel patients, respectively [23]. Similarly, VITAL-1 (*n* = 626) was also terminated early following a futility analysis showing that GVAX was unlikely to meet its primary endpoint of improved overall survival [24]. Despite these disappointing phase III results of GVAX, this immune-based agent is currently being trialled in combination with other immunotherapies, for example, Ipilimumab for PCa [76].

## 8. A Perspective on the Future of Immunotherapy for PCa

Since ADT plays a central role in the treatment of high-risk early PCa and recurrent or metastatic disease, it is crucial that immune-based therapies are compatible and, preferably, show synergistic activity. There is already preclinical and clinical evidence suggesting that ADT augments antitumour immunity through enhancement of T cell activation and trafficking to tumours [28, 34, 77, 78]. In addition, a reduction in tumour burden achieved through ADT (or surgery, chemotherapy, or other agents) may increase tumour sensitivity to immune-mediated destruction, as tumours can be highly immunosuppressive [79]. Moreover, the immune system is more likely to eliminate residual disease/low tumour volume than bulky lesions, a concept supported by the trend of longer survival time by sipuleucel-T or Prostvac-VF in patients with lower tumour burden [22, 46]. Recently, a phase II trial comprising nonmetastatic CRPC patients reported that Prostvac-VF, used in combination with the AR antagonist flutamide, was associated with longer median time to progression as compared to flutamide alone (233 days versus 85 days, resp.) [80], highlighting the potential of this combined approach. In terms of treatment sequencing, two phase II clinical trials provided early evidence that administration of ADT (nilutamide) after the immunological agent is associated with longer median survival [81] and median time to treatment failure [82], as compared to the reverse sequence. A possible explanation is that the immune modulatory effects of ADT augmented the antitumour immune responses initiated by Prostvac-VF. The optimal timing and sequence of Sipuleucel-T with novel hormone therapies such as abiraterone and enzalutamide are currently being investigated in phase II and III clinical trials, with the results being much anticipated [37, 83]. Similarly, the development of PD1 and PDL1 immune checkpoint inhibitors has attracted much interest [63]. Whilst occasional PCa patients have been enrolled in the phase I trials of these compounds, there has been no particular signal of activity in CRPC to date. Nevertheless, learning to harness PD1 and PDL1 inhibitors into the therapeutic armamentarium for CRPC would seem to be a priority.

Combination immunotherapy is another rational strategy to substantially enhance clinical outcomes by employing different agents that work via varied mechanisms of action and are potentially synergistic. A strategy that is receiving increasing attention is the combination of ipilimumab with Prostvac-VF. The goal is to reduce or eliminate regulatory elements during the induction and/or effector phases of antitumour immunity (ipilimumab), to further boost responses to the target antigen PSA (Prostvac-VF). A recent phase I trial of ipilimumab/Prostvac-VF combination reported encouraging results, with men treated with both agents having an overall survival of approximately 34 months. This compares favourably with historical controls of previous trials which used vaccine alone (26 months) [62, 84]. Moreover, there was evidence for antigen cascade induction, that is, immune responses developed against antigens not encoded in the vaccine, and that the combination did not seem to exacerbate the immune-related adverse events associated with ipilimumab. There are also trials now investigating the combination of GVAX with ipilimumab [76, 85]. While GVAX as a monotherapy had limited efficacy in phase III trials, it is an attractive candidate for combined usage with ipilimumab as the latter may augment immune responses to multiple tumour antigens. A recent phase I trial of ipilimumab and GVAX was well tolerated, and 25% of patients exhibited decline in PSA levels [85].

Chemoimmunotherapy is also a potentially useful approach for CRPC. In the past, chemotherapy was thought to compromise immune responses by way of lymphocyte lysis as a bystander effect of its mode of mechanism on actively dividing cancer cells. However, there is growing evidence that chemotherapy can induce immune modulatory effects that could facilitate the induction of antitumour immunity. For example, docetaxel has been reported to increase the production of proinflammatory cytokines [86], cyclophosphamide to decrease Tregs [87], cisplatin to upregulate the apoptosis ligand Fas [88], and doxorubicin to enhance cytotoxic T cell antitumour immunity [89]. However, the feasibility of chemoimmunotherapy for PCa remains to be determined, as early phase clinical trial results have so far been inconclusive. A phase II clinical trial involving men with metastatic CRPC reported that those given Prostvac-VF concurrently with docetaxel had longer progression-free survival than patients given the vaccine alone (3.2 months versus 1.8 months) [90], but this clearly needs to be validated in large prospective trials. On the other hand, a phase III trial that investigated the effects of GVAX plus docetaxel was terminated early following preliminary findings that the combination treatment was associated with shorter survival and had a higher death rate compared to the docetaxel plus prednisone control group [91]. To properly assess the clinical utility of chemoimmunotherapy, it is necessary to consider the optimal timing and scheduling of treatments.

## 9. Summary

While enzalutamide [12], abiratirone [10], and cabazitazel [8] have been shown to be efficacious in the postdocetaxel setting by prolonging survival, they are not curative; hence, novel therapies need to be integrated into treatment regimes to further improve patient outcome. Immunotherapy has the potential to meet this need, following the demonstration that some approaches can improve patient survival [19] and that other immune therapies may synergise with ADT [80] or with each other in their anticancer activity. The realisation that PCa is inherently immunogenic and, more importantly, can be targeted by the immune system through immune modulatory agents is driving intense research in this field. Various approaches are being explored, although only dendritic cell-based vaccines, immune checkpoint inhibition, viral vector, and tumour-based vaccines have reached phase III clinical trial endpoints to date. Key questions remain, including the mode of achieving immune modulation (vaccine? immune suppression modulation? TAAs?), timing of immune therapy according to stage of disease, sequence of treatments, and which candidate treatments might best be paired with immune therapy.

## Figures and Tables

**Table 1:** Key clinical trials based on the 4 selected immunotherapies for PCa.

Drug	Trial design	Number of patients	Phase	Key finding	Reference
Sipuleucel-T	Randomized, double blind, placebo controlled trial for asymptomatic metastatic CRPC	127	III	Improved OS by sipuleucel-T compared to placebo (25.9 versus 21.4 months)	[17]
	Randomized, double blind, placebo controlled trial for asymptomatic metastatic CRPC	98	III	Improved OS by sipuleucel-T compared to placebo (19 versus 15.7 months)	[18]
	Randomized, double blind, placebo controlled trial for asymptomatic metastatic CRPC	512	III	Improved OS by sipuleucel-T compared to placebo (25.8 versus 21.7 months)	[19]

Ipilimumab	Randomized, double blind, placebo-controlled trial for metastatic CRPC after docetaxel	799	III	No difference in OS between the 2 groups, but trend of improved PFS rate by ipilimumab at 6 months (30.7% versus 18.1%)	[20]

Prostvac-VF	Randomized placebo-controlled trial of Prostvac-VF for metastatic CRPC	125	II	Improved OS by Prostvac-VF compared to control vector placebo (25.1 versus 16.6 months)	[21]
	Nonrandomized trial for chemotherapy-naive CRPC	32	II	Improved OS by Prostvac-VF compared to historical controls (Halabi nomogram): (26.6 versus 17.4 months)	[22]

GVAX	Randomized trial of GVAX with docetaxel versus docetaxel with prednisone in taxane-naïve patients with symptomatic CRPC	408	III	Trial terminated early due to excess deaths in GVAX plus docetaxel group compared to control (docetaxel plus prednisone) (67 versus 47), and shorter median OS (12.2 versus 14.1 months).	[23]
	Randomized trial of GVAX with docetaxel versus docetaxel with prednisone in taxane-naïve patients with asymptomatic CRPC	626	III	Trial terminated early based on futility analysis showing <30% chance of meeting primary endpoint (improved OS)	[24]

## References

[1] Jemal A, Bray F, Center MM, Ferlay J, Ward E, Forman D (2011). Global cancer statistics. *CA: A Cancer Journal for Clinicians*.

[2] Westdorp H, Skold AE, Snijer BA (2014). Immunotherapy for prostate cancer: lessons from responses to tumor-associated antigens. *Frontiers in Immunology*.

[3] Toren PJ, Gleave ME (2013). Evolving landscape and novel treatments in metastatic castrate-resistant prostate cancer. *Asian Journal of Andrology*.

[4] Attard G, Richards J, de Bono JS (2011). New strategies in metastatic prostate cancer: targeting the androgen receptor signaling pathway. *Clinical Cancer Research*.

[5] Schröder F, Crawford ED, Axcrona K, Payne H, Keane TE (2012). Androgen deprivation therapy: past, present and future. *BJU International*.

[6] Petrylak DP, Tangen CM, Hussain MHA (2004). Docetaxel and estramustine compared with mitoxantrone and prednisone for advanced refractory prostate cancer. *The New England Journal of Medicine*.

[7] Tannock IF, de Wit R, Berry WR (2004). Docetaxel plus prednisone or mitoxantrone plus prednisone for advanced prostate cancer. *The New England Journal of Medicine*.

[8] de Bono JS, Oudard S, Ozguroglu M (2010). Prednisone plus cabazitaxel or mitoxantrone for metastatic castration-resistant prostate cancer progressing after docetaxel treatment: a randomised open-label trial. *The Lancet*.

[9] Locke JA, Guns ES, Lubik AA (2008). Androgen Levels increase by intratumoral de novo steroidogenesis during progression of castration-resistant prostate cancer. *Cancer Research*.

[10] de Bono JS, Logothetis CJ, Molina A (2011). Abiraterone and increased survival in metastatic prostate cancer. *New England Journal of Medicine*.

[11] Attard G, Reid AHM, A'Hern R (2009). Selective inhibition of CYP17 with abiraterone acetate is highly active in the treatment of castration-resistant prostate cancer. *Journal of Clinical Oncology*.

[12] Scher HI, Fizazi K, Saad F (2012). Increased survival with enzalutamide in prostate cancer after chemotherapy. *The New England Journal of Medicine*.

[13] Kantoff P, Higano CS (2012). Integration of immunotherapy into the management of advanced prostate cancer. *Urologic Oncology*.

[14] Vesely MD, Kershaw MH, Schreiber RD, Smyth MJ (2011). Natural innate and adaptive immunity to cancer. *Annual Review of Immunology*.

[15] Vivier E, Ugolini S, Blaise D, Chabannon C, Brossay L (2012). Targeting natural killer cells and natural killer T cells in cancer. *Nature Reviews Immunology*.

[16] Palucka K, Banchereau J (2012). Cancer immunotherapy via dendritic cells. *Nature Reviews Cancer*.

[17] Small EJ, Schellhammer PF, Higano CS (2006). Placebo-controlled phase III trial of immunologic therapy with Sipuleucel-T (APC8015) in patients with metastatic, asymptomatic hormone refractory prostate cancer. *Journal of Clinical Oncology*.

[18] Higano CS, Schellhammer PF, Small EJ (2009). Integrated data from 2 randomized, double-blind, placebo-controlled, phase 3 trials of active cellular immunotherapy with sipuleucel-T in advanced prostate cancer. *Cancer*.

[19] Kantoff PW, Higano CS, Shore ND (2010). Sipuleucel-T immunotherapy for castration-resistant prostate cancer. *The New England Journal of Medicine*.

[20] Kwon ED, Drake CG, Scher HI (2014). Ipilimumab versus placebo after radiotherapy in patients with metastatic castration-resistant prostate cancer that had progressed after docetaxel chemotherapy (CA184-043): a multicentre, randomised, double-blind, phase 3 trial. *The Lancet Oncology*.

[21] Kantoff PW, Schuetz TJ, Blumenstein BA (2010). Overall survival analysis of a phase II randomized controlled trial of a poxviral-based PSA-targeted immunotherapy in metastatic castration-resistant prostate cancer. *Journal of Clinical Oncology*.

[22] Gulley JL, Arlen PM, Madan RA (2010). Immunologic and prognostic factors associated with overall survival employing a poxviral-based PSA vaccine in metastatic castrate-resistant prostate cancer. *Cancer Immunology, Immunotherapy*.

[23] Small TD, Gerritsen WR, Rolland F A phase III trial of GVAX immunotherapy for prostate cancer in combination with docetaxel vs docetaxel plus prednisone in symptomatic, castration-resistant prostate cancer (CRPC).

[24] Higano FS C, Somer B, Curti B A phase III trial of GVAX immunotherapy for prostate cancer versus docetaxel plus prednisone in asymptomatic, castration-resistant prostate cancer (CRPC).

[25] Vesalainen S, Lipponen P, Talja M, Syrjanen K (1994). Histological grade, perineural infiltration, tumour-infiltrating lymphocytes and apoptosis as determinants of long-term prognosis in prostatic adenocarcinoma. *European Journal of Cancer A*.

[26] Hussein MA, AL-Assiri M, Musalam AO (2009). Phenotypic characterization of the infiltrating immune cells in normal prostate, benign nodular prostatic hyperplasia and prostatic adenocarcinoma. *Experimental and Molecular Pathology*.

[27] McArdle PA, Canna K, McMillan DC, McNicol A-, Campbell R, Underwood MA (2004). The relationship between T-lymphocyte subset infiltration and survival in patients with prostate cancer. *The British Journal of Cancer*.

[28] Gannon PO, Poisson AO, Delvoye N, Lapointe R, Mes-Masson A, Saad F (2009). Characterization of the intra-prostatic immune cell infiltration in androgen-deprived prostate cancer patients. *Journal of Immunological Methods*.

[29] Davidsson S, Ohlson A, Andersson S (2013). CD4 helper T cells, CD8 cytotoxic T cells, and FOXP3^+^ regulatory T cells with respect to lethal prostate cancer. *Modern Pathology*.

[30] Lanciotti M, Masieri L, Raspollini MR (2014). The role of M1 and M2 macrophages in prostate cancer in relation to extracapsular tumor extension and biochemical recurrence after radical prostatectomy. *BioMed Research International*.

[31] Shimura S, Yang G, Ebara S, Wheeler TM, Frolov A, Thompson TC (2000). Reduced infiltration of tumor-associated macrophages in human prostate cancer: association with cancer progression. *Cancer Research*.

[32] Nonomura N, Takayama H, Nakayama M (2011). Infiltration of tumour-associated macrophages in prostate biopsy specimens is predictive of disease progression after hormonal therapy for prostate cancer. *BJU International*.

[33] Miller AM, Pisa P (2007). Tumor escape mechanisms in prostate cancer. *Cancer Immunology, Immunotherapy*.

[34] Mercader M, Bodner BK, Moser MT (2001). T cell infiltration of the prostate induced by androgen withdrawal in patients with prostate cancer. *Proceedings of the National Academy of Sciences of the United States of America*.

[35] Wesley JD, Whitmore J, Trager J, Sheikh N (2012). An overview of sipuleucel-T: autologous cellular immunotherapy for prostate cancer. *Human Vaccines & Immunotherapeutics*.

[36] Sheikh NA, Petrylak D, Kantoff PW (2013). Sipuleucel-T immune parameters correlate with survival: an analysis of the randomized phase 3 clinical trials in men with castration-resistant prostate cancer. *Cancer Immunology, Immunotherapy*.

[37] Paller CJ, Antonarakis ES (2012). Sipuleucel-T for the treatment of metastatic prostate cancer: promise and challenges. *Human Vaccines & Immunotherapeutics*.

[38] Gunia S, Koch S, May M, Dietel M, Erbersdobler A (2009). Expression of prostatic acid phosphatase (PSAP) in transurethral resection specimens of the prostate is predictive of histopathologic tumor stage in subsequent radical prostatectomies. *Virchows Archiv*.

[39] Graddis TJ, McMahan CJ, Tamman J, Page KJ, Trager JB (2011). Prostatic acid phosphatase expression in human tissues. *International Journal of Clinical and Experimental Pathology*.

[40] Fang LC, Dattoli M, Taira A, True L, Sorace R, Wallner K (2008). Prostatic acid phosphatase adversely affects cause-specific survival in patients with intermediate to high-risk prostate cancer treated with brachytherapy. *Urology*.

[41] Arellano M, Lonial S (2008). Clinical uses of GM-CSF, a critical appraisal and update. *Biologics: Targets and Therapy*.

[42] Laus R, Yang DM, Ruegg CL (2001). Dendritic cell immunotherapy of prostate cancer: preclinical models and early clinical experience. *Cancer Research, Therapy and Control*.

[43] Sims RB (2012). Development of sipuleucel-T: autologous cellular immunotherapy for the treatment of metastatic castrate resistant prostate cancer. *Vaccine*.

[44] Small E, Fratesi P, Reese D (2000). Immunotherapy of hormone-refractory prostate cancer with antigen-loaded dendritic cells. *Journal of Clinical Oncology*.

[45] Burch PA, Breen JK, Buckner JC (2000). Priming tissue-specific cellular immunity in a phase I trial of autologous dendritic cells for prostate cancer. *Clinical Cancer Research*.

[46] Schellhammer PF, Chodak G, Whitmore JB, Sims R, Frohlich MW, Kantoff PW (2013). Lower baseline prostate-specific antigen is associated with a greater overall survival benefit from sipuleucel-T in the immunotherapy for prostate adenocarcinoma treatment (IMPACT) trial. *Urology*.

[47] Chen L, Flies DB (2013). Molecular mechanisms of T cell co-stimulation and co-inhibition. *Nature Reviews Immunology*.

[48] Wing K, Onishi Y, Prieto-Martin P (2008). CTLA-4 control over Foxp3+ regulatory T cell function. *Science*.

[49] Kwon ED, Hurwitz AA, Foster BA (1997). Manipulation of T cell costimulatory and inhibitory signals for immunotherapy of prostate cancer. *Proceedings of the National Academy of Sciences of the United States of America*.

[50] Greenberg NM, DeMayo F, Finegold MJ (1995). Prostate cancer in a transgenic mouse. *Proceedings of the National Academy of Sciences of the United States of America*.

[51] Gingrich JR, Barrios RJ, Morton RA (1996). Metastatic prostate cancer in a transgenic mouse. *Cancer Research*.

[52] Kwon ED, Foster BA, Hurwitz AA (1999). Elimination of residual metastatic prostate cancer after surgery and adjunctive cytotoxic T lymphocyte-associated antigen 4 (CTLA-4) blockade immunotherapy. *Proceedings of the National Academy of Sciences of the United States of America*.

[53] Sondak VK, Smalley KSM, Kudchadkar R, Grippon S, Kirkpatrick P (2011). Ipilimumab. *Nature Reviews Drug Discovery*.

[54] Hodi FS, O'Day SJ, McDermott DF (2010). Improved survival with ipilimumab in patients with metastatic melanoma. *The New England Journal of Medicine*.

[55] Tomasini P, Khobta N, Greillier L, Barlesi F (2012). Ipilimumab: Its potential in non-small cell lung cancer. *Therapeutic Advances in Medical Oncology*.

[56] Yang JC, Hughes M, Kammula U (2007). Ipilimumab (anti-CTLA4 antibody) causes regression of metastatic renal cell cancer associated with enteritis and hypophysitis. *Journal of Immunotherapy*.

[57] Tse BW, Collins A, Oehler MK (2014). Antibody-based immunotherapy for ovarian cancer: where are we at?. *Annals of Oncology*.

[58] Hodi FS, Mihm MC, Soiffer RJ (2003). Biologic activity of cytotoxic T lymphocyte-associated antigen 4 antibody blockade in previously vaccinated metastatic melanoma and ovarian carcinoma patients. *Proceedings of the National Academy of Sciences of the United States of America*.

[59] Small EJ, Tchekmedyian NS, Rini BI, Fong L, Lowy I, Allison JP (2007). A pilot trial of CTLA-4 blockade with human anti-CTLA-4 in patients with hormone-refractory prostate cancer. *Clinical Cancer Research*.

[60] Slovin SF, Higano CS, Hamid O (2013). Ipilimumab alone or in combination with radiotherapy in metastatic castration-resistant prostate cancer: results from an open-label, multicenter phase I/II study. *Annals of Oncology*.

[61] Arlen PM, Kaufman HL, DiPaola RS (2005). Pox viral vaccine approaches. *Seminars in Oncology*.

[62] Madan RA, Bilusic M, Heery C, Schlom J, Gulley JL (2012). Clinical evaluation of TRICOM vector therapeutic cancer vaccines. *Seminars in Oncology*.

[63] Drake CG (2010). Prostate cancer as a model for tumour immunotherapy. *Nature Reviews Immunology*.

[64] Hodge JW, Grosenbach DW, Aarts WM, Poole DJ, Schlom J (2003). Vaccine therapy of established tumors in the absence of autoimmunity. *Clinical Cancer Research*.

[65] DiPaola RS, Plante M, Kaufman H (2006). A phase I trial of pox PSA vaccines (PROSTVAC-VF) with B7-1, ICAM-1, and LFA-3 co-stimulatory molecules (TRICOM) in patients with prostate cancer. *Journal of Translational Medicine*.

[66] Sanda MG, Smith DC, Charles LG (1999). Recombinant vaccinia-PSA (PROSTVAC) can induce a prostate-specific immune response in androgen-modulated human prostate cancer. *Urology*.

[67] Kaufman HL, Wang W, Manola J (2004). Phase II randomized study of vaccine treatment of advanced prostate cancer (E7897): a trial of the Eastern Cooperative Oncology Group. *Journal of Clinical Oncology*.

[68] Gulley JL, Madan RA, Tsang KY (2014). Immune impact induced by PROSTVAC (PSA-TRICOM), a therapeutic vaccine for prostate cancer. *Cancer Immunology Research*.

[69] Ward JE, McNeel DG (2007). GVAX: an allogeneic, whole-cell, GM-CSF-secreting cellular immunotherapy for the treatment of prostate cancer. *Expert Opinion on Biological Therapy*.

[70] Dranoff G, Jaffee E, Lazenby A (1993). Vaccination with irradiated tumor cells engineered to secrete murine granulocyte-macrophage colony-stimulating factor stimulates potent, specific, and long-lasting anti-tumor immunity. *Proceedings of the National Academy of Sciences of the United States of America*.

[71] Sanda MG, Ayyagari SR, Jaffee EM (1994). Demonstration of a rational strategy for human prostate cancer gene therapy. *Journal of Urology*.

[72] Simons JW, Carducci MA, Mikhak B (2006). Phase I/II trial of an allogeneic cellular immunotherapy in hormone-naïve prostate cancer. *Clinical Cancer Research*.

[73] Small EJ, Sacks N, Nemunaitis J (2007). Granulocyte macrophage colony-stimulating factor-secreting allogeneic cellular immunotherapy for hormone-refractory prostate cancer. *Clinical Cancer Research*.

[74] Higano CS, Corman JM, Smith DC (2008). Phase 1/2 dose-escalation study of a GM-CSF-secreting, allogeneic, cellular immunotherapy for metastatic hormone-refractory prostate cancer. *Cancer*.

[75] Drake CG (2009). Immunotherapy for prostate cancer: walk, don’t run. *Journal of Clinical Oncology*.

[76] Schweizer MT, Drake CG (2014). Immunotherapy for prostate cancer: recent developments and future challenges. *Cancer and Metastasis Reviews*.

[77] Drake CG, Doody ADH, Mihalyo MA (2005). Androgen ablation mitigates tolerance to a prostate/prostate cancer-restricted antigen. *Cancer Cell*.

[78] Morse MD, McNeel DG (2010). Prostate cancer patients on androgen deprivation therapy develop persistent changes in adaptive immune responses. *Human Immunology*.

[79] Lindau D, Gielen P, Kroesen M, Wesseling P, Adema GJ (2013). The immunosuppressive tumour network: myeloid-derived suppressor cells, regulatory T cells and natural killer T cells. *Immunology*.

[80] Bilusic M, Gulley JL, Heery C (2011). A randomized phase II study of flutamide with or without PSA-TRICOM in nonmetastatic castration-resistant prostate cancer (CRPC). *Journal of Clinical Oncology*.

[81] Madan RA, Gulley JL, Schlom J (2008). Analysis of overall survival in patients with nonmetastatic castration-resistant prostate cancer treated with vaccine, nilutamide, and combination therapy. *Clinical Cancer Research*.

[82] Arlen PM, Gulley JL, Todd N (2005). Antiandrogen, vaccine and combination therapy in patients with nonmetastatic hormone refractory prostate cancer. *Journal of Urology*.

[83] Antonarakis ES, Drake CG (2012). Combining immunological and androgen-directed approaches: an emerging concept in prostate cancer immunotherapy. *Current Opinion in Oncology*.

[84] Madan RA, Mohebtash M, Arlen PM (2012). Ipilimumab and a poxviral vaccine targeting prostate-specific antigen in metastatic castration-resistant prostate cancer: a phase 1 dose-escalation trial. *The Lancet Oncology*.

[85] van den Eertwegh AJM, Versluis J, van den Berg HP (2012). Combined immunotherapy with granulocyte-macrophage colony-stimulating factor-transduced allogeneic prostate cancer cells and ipilimumab in patients with metastatic castration-resistant prostate cancer: a phase 1 dose-escalation trial. *The Lancet Oncology*.

[86] Chan OTM, Yang L (2000). The immunological effects of taxanes. *Cancer Immunology Immunotherapy*.

[87] Lutsiak MEC, Semnani RT, de Pascalis R, Kashmiri SVS, Schlom J, Sabzevari H (2005). Inhibition of CD4^+^25^+^ T regulatory cell function implicated in enhanced immune response by low-dose cyclophosphamide. *Blood*.

[88] Matsuzaki I, Suzuki H, Kitamura M, Minamiya Y, Kawai H, Ogawa J (2000). Cisplatin induces Fas expression in esophageal cancer cell lines and enhanced cytotoxicity in combination with LAK cells. *Oncology*.

[89] Mattarollo SR, Loi S, Duret H, Ma Y, Zitvogel L, Smyth MJ (2011). Pivotal role of innate and adaptive immunity in anthracycline chemotherapy of established tumors. *Cancer Research*.

[90] Arlen PM, Gulley JL, Parker C (2006). A randomized phase II study of concurrent docetaxel plus vaccine versus vaccine alone in metastatic androgen-independent prostate cancer. *Clinical Cancer Research*.

[91] Uhlman MA, Bing MT, Lubaroff DM (2014). Prostate cancer vaccines in combination with additional treatment modalities. *Immunologic Research*.

